# Tanshinone IIA suppresses the progression of atherosclerosis by inhibiting the apoptosis of vascular smooth muscle cells and the proliferation and migration of macrophages induced by ox-LDL

**DOI:** 10.1242/bio.024133

**Published:** 2017-04-15

**Authors:** Baocai Wang, Zhenwei Ge, Zhaoyun Cheng, Ziniu Zhao

**Affiliations:** Department of Cardiovascular Surgery, Henan Provincial People's Hospital, Zhengzhou 450003, People's Republic of China

**Keywords:** Atherosclerosis, Macrophages, ox-LDL, Tanshinone IIA, Vascular smooth muscle cells

## Abstract

The profound inhibitory effect of Tanshinone IIA (Tan IIA) on atherosclerosis has been demonstrated, whereas the latent mechanism is not completely cleared. This study aimed to investigate the cellular and molecular mechanisms underlying Tan IIA protecting against atherosclerosis. Oil Red O staining and ELISA assay showed that Tan IIA suppressed the progress of atherosclerosis and reduced the levels of inflammatory cytokines in serum of apolipoprotein E deficient (ApoE*^–/–^*) mice. Flow cytometry assay revealed that Tan IIA inhibited oxidized LDL (ox-LDL)-induced apoptosis of VSMCs. MTT and transwell assay indicated that Tan IIA suppressed the ox-LDL-stimulated proliferation and migration of RAW264.7 cells. Moreover, Tan IIA ameliorated inflammatory cytokine upregulation elicited by ox-LDL in RAW264.7 cells. Additionally, Tan IIA inhibited the apoptosis of VSMCs and decreased the levels of MMP-2, MMP-9 in ApoE^–/–^ mice. In conclusion, our study demonstrated Tan IIA suppressed the progression of atherosclerosis by inhibiting vascular inflammation, apoptosis of VSMCs and proliferation and migration of macrophages induced by ox-LDL.

## Abstract (precis)

**Summary:** Tanshinone IIA may become a new approach for the prevention of atherosclerosis progression through inhibiting the apoptosis of vascular smooth muscle cells and the proliferation and migration of macrophages.

## INTRODUCTION

Atherosclerosis is a well-known pathological manifestation of cardiovascular diseases belonging to chronic inflammatory disease, characterized by the formation of atherosclerotic plaques (Wang et al., 2013). Lesions occur mainly in large and medium elastic and muscular arteries and may result in ischemia of the heart, brain, and extremities, or stroke (Fenyo and Gafencu, 2013). Among numerous genetic and environmental causes, the deposition of modified low density lipoprotein (LDL), such as oxidized LDL (ox-LDL) (Steinberg et al., 1989), the recruitment of monocyte-derived macrophages at the arterial subendothelial spaces (Fantuzzi and Mazzone, 2007), and accumulation of vascular smooth muscle cells (VSMCs) (Gerthoffer, 2007) are the crucial elements resulting in the development of atherosclerotic lesion.

Ox-LDL has been reported in the development of atherosclerosis (Holvoet et al., 2004). Some biological processes contribute to the atherosclerotic plaque formation and progression, including smooth muscle cell migration and proliferation, macrophage foam cell formation, and altered expression of cytokines and growth factors (Pirillo et al., 2013). Ox-LDL significantly promotes VSMCs migration and proliferation in intimal area through activating the ERK1/2 signaling pathway transcription factors, which is a main feature of atherosclerotic lesions (Cutini and Massheimer, 2010; Lin et al., 2016; Yang et al., 2001). In macrophages, ox-LDL is capable of targeting several scavenger receptors and induces production of proinflammatory cytokines such as tumor necrosis factor (TNF)-α, oxidative stress, and enhances chemotaxis (Sun et al., 2009).

In atherosclerosis, VSMCs are involved in reconstruction of the arterial wall in order to maintain blood flow in affected vessels due to atherosclerotic alteration (Chistiakov et al., 2015). VSMCs exist in the media in a quiescent state in the normal blood vessel, but they migrate into the intima following injury, and their over proliferation can cause the constraint of normal blood flow (Johnson et al., 2011; Murry et al., 1997). VSMCs apoptosis takes place in many arterial diseases, such as angioplasty restenosis, aneurysm formation and atherosclerosis, and is connect to atherosclerotic plaque rupture (Clarke et al., 2006; Su et al., 2015). Macrophages from circulating blood monocytes can be involved in inflammatory response (Murray and Wynn, 2011). Macrophages absorb lots of ox-LDL under the intima and then become foam cells, which is one of the early signs of atherosclerotic lesions (Fenyo and Gafencu, 2013).

Tanshinone IIA (Tan IIA), a kind of traditional Chinese medicine, is one of the main fat-soluble ingredients of *Radix Salvia miltiorrhiza* (also known as Danshen), and a proverbial flavonoid that has demonstrated a valid antioxidant for protecting against atherosclerosis (You et al., 2012; Zheng et al., 2014). Previous reports show that Tan IIA inhibits the atherosclerotic lesion in rat and rabbit through suppressing the oxidative stress and inflammation (Fang et al., 2008; Tang et al., 2011). Chen and Xu (2014)  indicate that Tan IIA inhibits atherosclerosis by regulating the apoptosis and expression of inflammatory factors in atherosclerosis plaques. Nevertheless, the precise cellular and molecular mechanisms by which Tan IIA protect against atherosclerosis remained unclear. Tan IIA is able to suppress the oxidative modification of LDL into ox-LDL both *in vivo* and *in vitro* (Niu et al., 2000; Tang et al., 2007). Thus, it is presumed that Tan IIA exerts the anti-atherosclerotic effect likely through inhibiting the effect of ox-LDL on atherosclerosis.

In this study, the role of Tan IIA was explored in atherosclerotic lesion in ApoE*^−/−^* mice. The functional effect of Tan IIA on VSMCs and RAW264.7 cells was further investigated to study the molecular mechanisms of Tan IIA protecting against atherosclerosis.

## RESULTS

### Tan IIA inhibited atherosclerosis in ApoE^–/–^ mice

**Fig. 1.**
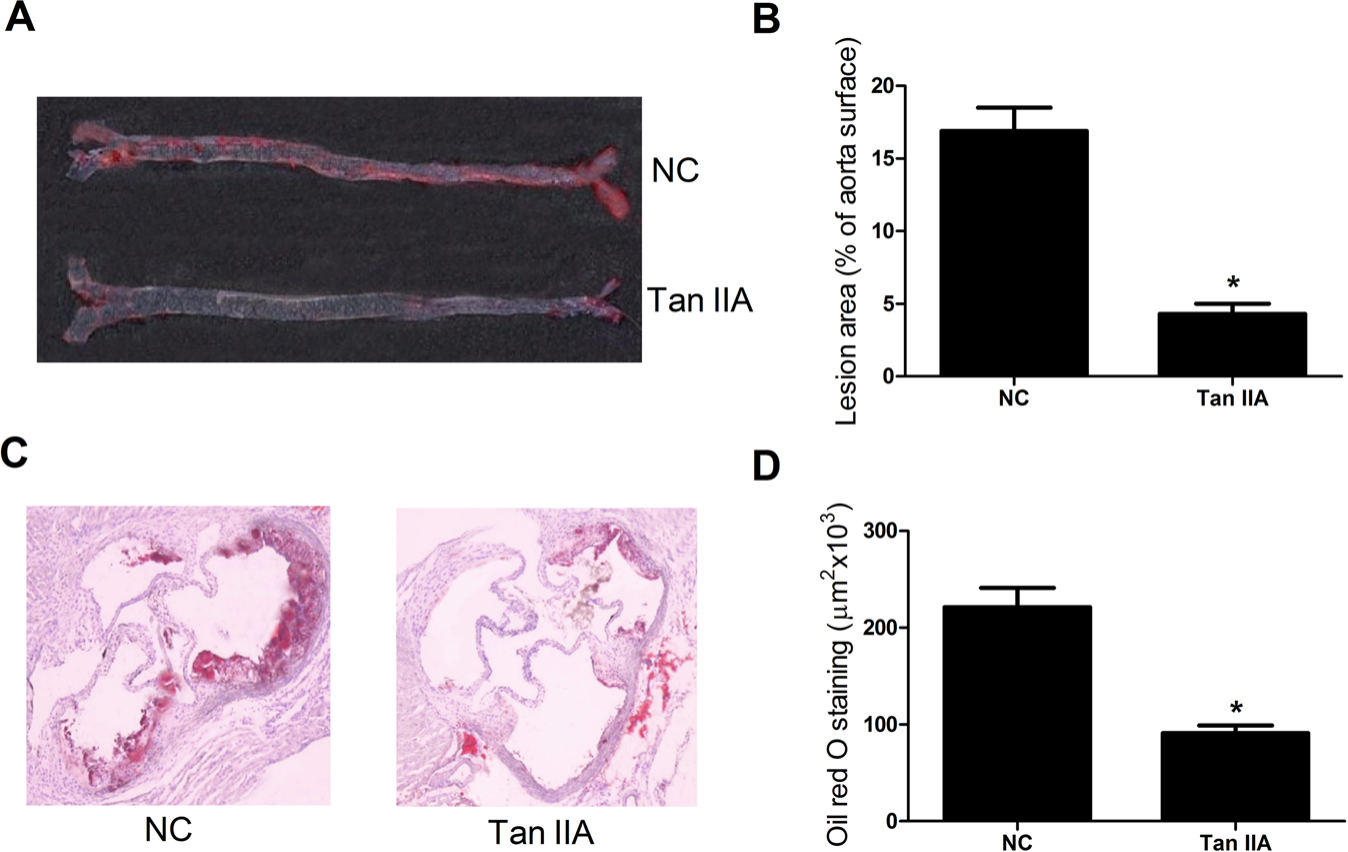
**Effects of Tan IIA on atherosclerosis in ApoE*^−/−^* mice.** ApoE^−/−^ mice at 6 weeks were gavaged with Tan IIA (30 mg/kg) for 20 weeks and then sacrificed. (A) Representative photographs indicating the atherosclerotic plaque in aortas from ApoE*^–/–^* mice with Oil Red O. (B) The atherosclerotic lesion area in the Tan IIA group and control group. (C) Representative photomicrographs of the aortic root from ApoE*^−/−^* mice stained with Oil Red O. (D) The atherosclerotic lesion was quantified as the fraction area in Tan IIA group and control group. In B and D, *n*=8, *P<0.05, error bars indicate that data were expressed as the mean±s.d.

To test whether Tan IIA alleviated atherosclerosis, the plaque area of aortic arches and staining area of aortic roots were estimated using Oil Red O staining. It was found that the aortic arches plaque area (%) (Fig. 1A,B) and aortic root slice staining area (Fig. 1C,D) of ApoE^–/–^ mice fed with Tan IIA were obviously reduced in comparison with the control group.

### Tan IIA decreased the levels of inflammatory cytokines in serum of ApoE^–/–^ mice

**Fig. 2.**
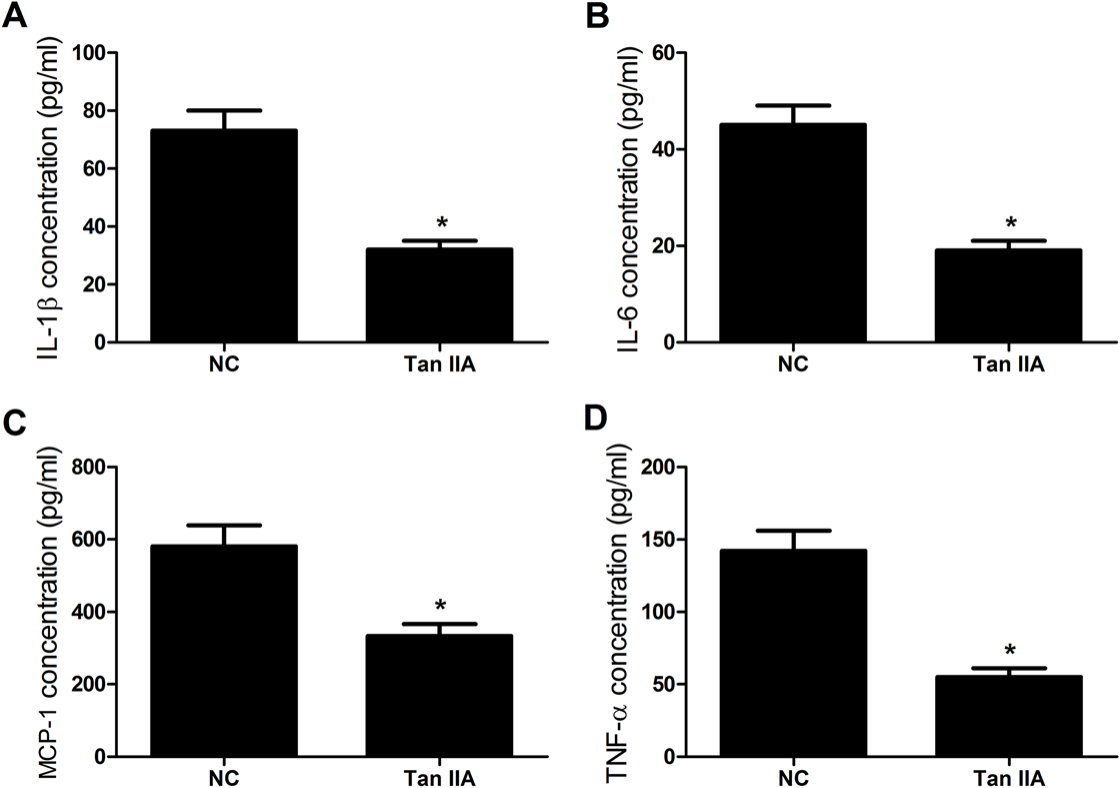
**Tan IIA suppresses expression of inflammatory cytokines in serum of ApoE*^–/–^* mice.** ApoE^−/−^ mice at 6 weeks were gavaged with Tan IIA (30 mg/kg) for 20 weeks and then sacrificed. (A-D) The concentrations of IL-1β, IL-6, MCP-1, and TNF-α in serum of ApoE*^–/–^* mice treated with Tan IIA were significantly decreased by ELISA assay. *n*=8, *P<0.05, error bars indicate that data were expressed as the mean±s.d.

The contents of four main inflammatory cytokines (IL-1β, IL-6, MCP-1, and TNF-α) in serum of ApoE*^–/–^* mice were detected by ELISA. As shown in Fig. 2A-D, the serum levels of IL-1β, IL-6, MCP-1, and TNF-α in ApoE^–/–^ mice treated with Tan IIA were dramatically reduced compared with the control group. These results indicated that Tan IIA exerted an anti-inflammatory role in atherosclerosis.

### Tan IIA attenuated ox-LDL-induced apoptosis of VSMCs

**Fig. 3.**
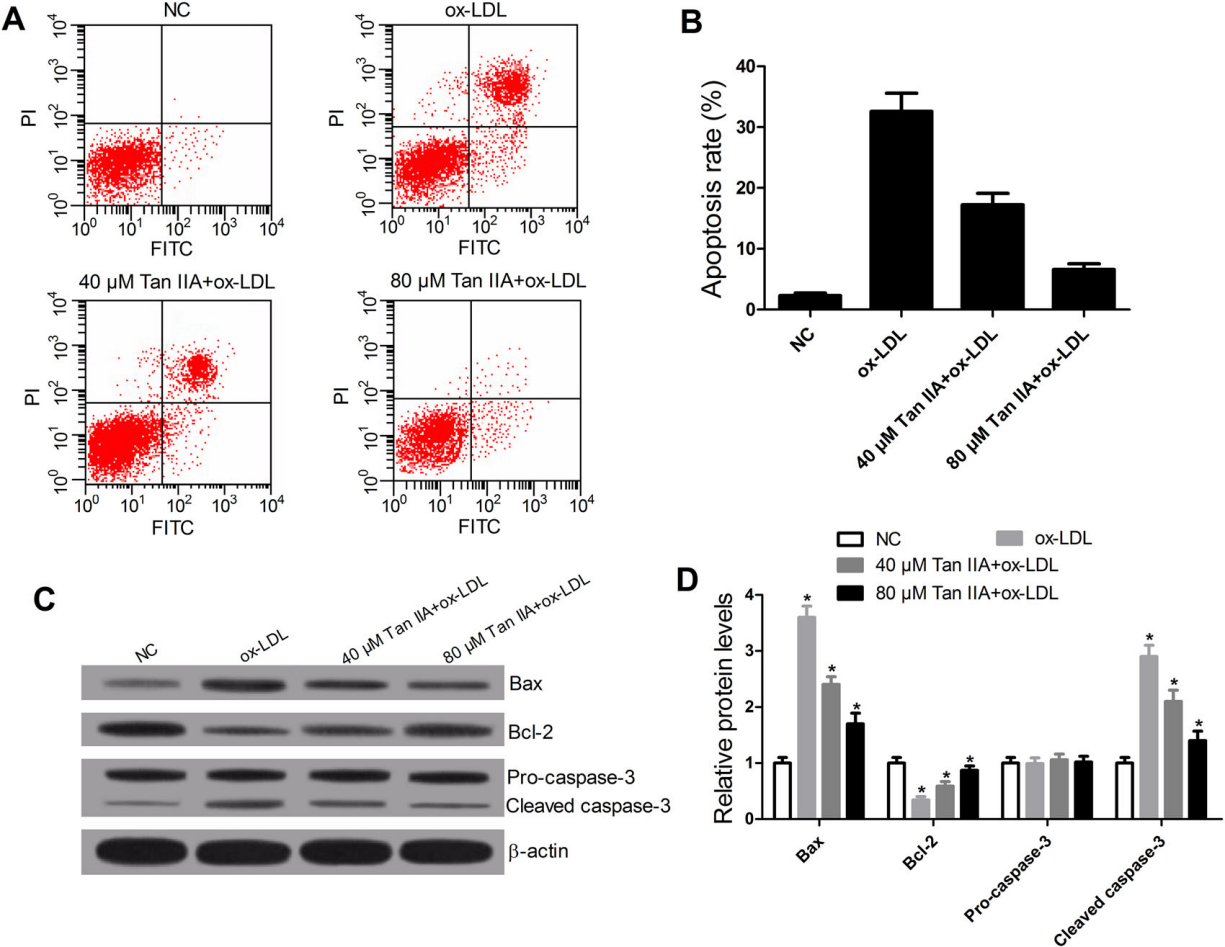
**Tan IIA inhibits ox-LDL-induced apoptosis of VSMCs through regulating the apoptosis-related protein levels.** VSMCs were treated with ox-LDL (50 μg/ml) or ox-LDL+Tan IIA (40 or 80 μM) for 24 h. (A,B) Flow cytometry showed that Tan IIA reversed ox-LDL-induced apoptosis in VSMCs. (C,D) Western blot displayed that Tan IIA overturned the effects of ox-LDL on the expression of Bcl-2, Bax and cleaved caspase-3. *n*=4, *P<0.05, error bars in B and D indicate that data were expressed as the mean±s.d.

The effects of Tan IIA on ox-LDL-stimulated VSMCs responses were investigated. The role of ox-LDL in apoptosis of VSMCs was detected by flow cytometry assay. When VSMCs were treated with ox-LDL, an obvious increase in the rate of apoptosis was observed. However, this effect was significantly decreased by Tan IIA treatment (Fig. 3A,B). Further apoptosis-related protein levels (Bax, Bcl-2, pro-caspase-3, and Cleaved caspase-3) were determined by western blot. The data showed that ox-LDL notably reduced the expression of Bcl-2 and increased the levels of Bax and cleaved caspase-3, whereas Tan IIA reversed the effects of ox-LDL (Fig. 3C,D). These results demonstrated that Tan IIA decreased the apoptosis induced by ox-LDL in VSMCs.

### Tan IIA inhibited the ox-LDL-induced proliferation and migration of RAW264.7 cells

**Fig. 4.**
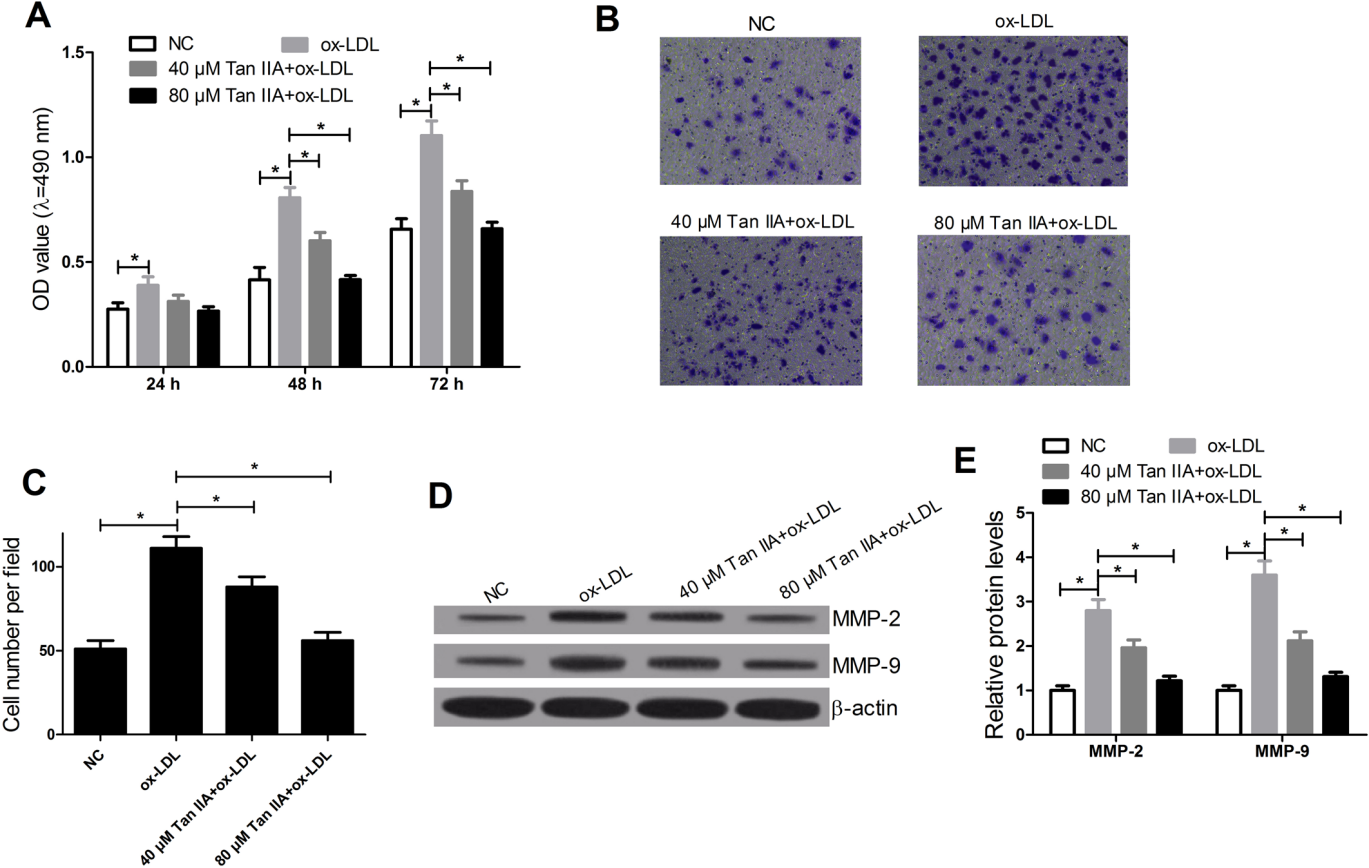
**Tan IIA inhibited the ox-LDL-induced proliferation and migration of RAW264.7 cells.** RAW264.7 cells were treated with ox-LDL (50 μg/ml) or Tan IIA (40 or 80 μM). (A) MTT was used to measure the effect of ox-LDL and Tan IIA on cell proliferation. (B,C) Transwell chamber was performed to detect the effect of ox-LDL and Tan IIA on cell migration after treatment with ox-LDL or (ox-LDL+Tan IIA) for 24 h. (D,E) Western blots were carried out to determine the levels of MMP-9 and MMP-2 after treatment with ox-LDL or (ox-LDL+Tan IIA) for 24 h in RAW264.7 cells. *n*=4, *P<0.05, error bars in A, C and E indicate that data were expressed as the mean±s.d.

The effects of Tan IIA on ox-LDL-induced RAW264.7 cells responses were determined by MTT assay and migration assay. The results showed that ox-LDL evidently promoted RAW264.7 cell proliferation, whereas the stimulative effect of ox-LDL on RAW264.7 cell proliferation was reversed by Tan IIA (Fig. 4A); and then the effects of Tan IIA on ox-LDL-induced cell migration were detected by cell migration assay. The data demonstrated that the migration ability of RAW264.7 cells treated with ox-LDL was drastically increased, whereas Tan IIA abated the positive effect of ox-LDL on RAW264.7 cell migration (Fig. 4B,C). In addition, the levels of MMP-9 and MMP-2 in RAW264.7 cells were detected by western blot. The data indicated that protein levels of MMP-9 and MMP-2 were markedly up-regulated by ox-LDL, while Tan IIA abolished the positive effect of ox-LDL on regulating expression of MMP-9 and MMP-2 (Fig. 4 D,E). These results suggested that Tan IIA suppressed the proliferation and migration elicited by ox-LDL in RAW264.7 cells.

### Tan IIA ameliorates inflammatory cytokine upregulation in ox-LDL-stimulated RAW264.7 cells

**Fig. 5.**
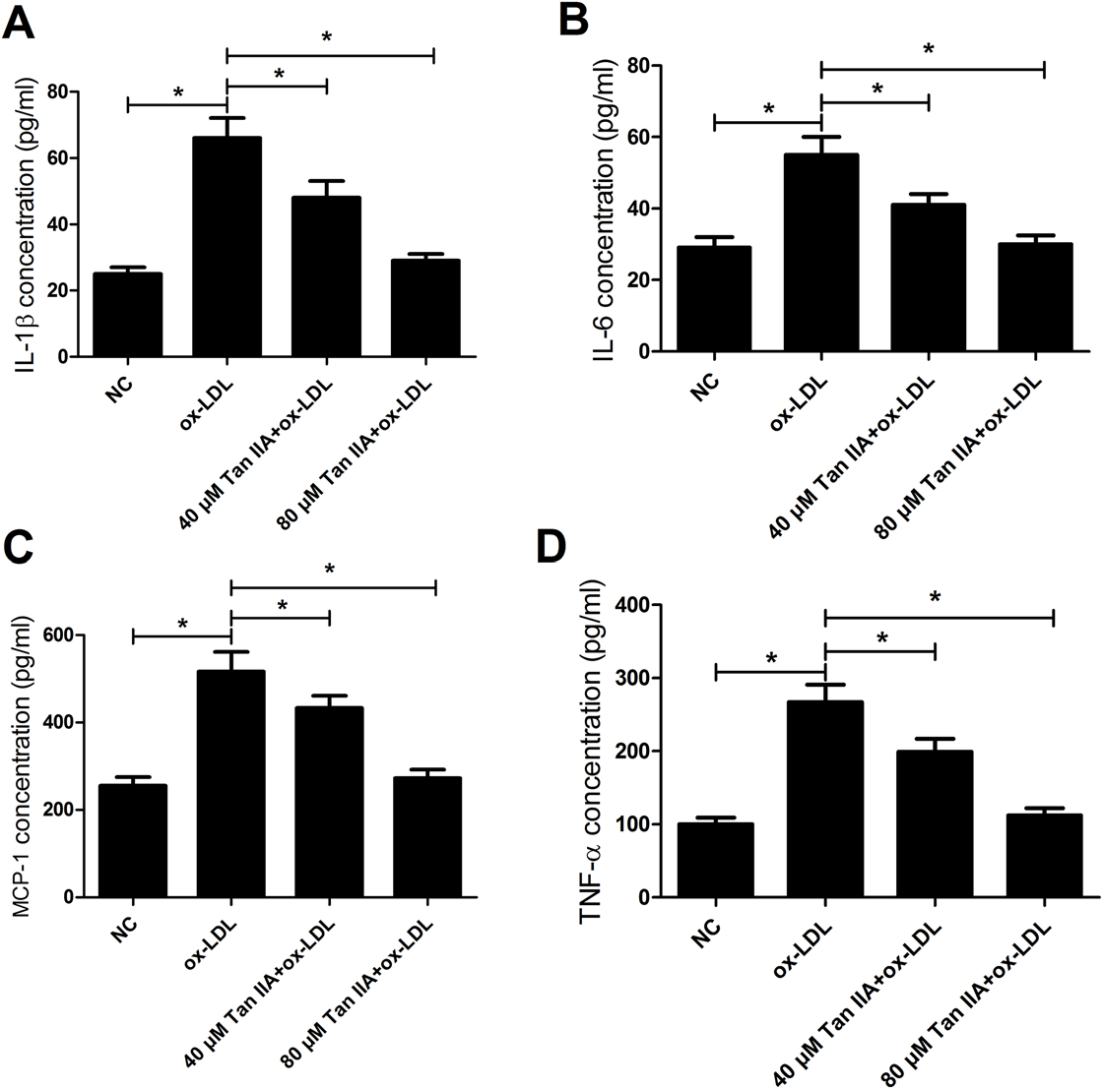
**Tan IIA ameliorates inflammatory cytokine upregulation in ox-LDL-stimulated RAW264.7 cells.** RAW264.7 cells were treated with ox-LDL (50 μg/ml) or (ox-LDL+Tan IIA) (40 or 80 μM) for 24 h. (A-D) The concentrations of inflammatory cytokines (IL-1β, IL-6, MCP-1, and TNF-α) in RAW264.7 cells treated with ox-LDL or (ox-LDL+Tan IIA) were detected by ELISA. *n*=4, *P<0.05, error bars indicate that data were expressed as the mean±s.d.

The concentrations of four main inflammatory cytokines (IL-1β, IL-6, MCP-1, and TNF-α) in RAW264.7 cells were detected by ELISA. As shown in Fig. 5A-D, ox-LDL led to a prominent augment in the levels of IL-1β, IL-6, MCP-1, and TNF-α in RAW264.7 cells compared with the control group, while Tan IIA treatment inhibited this effect. These results indicated that Tan IIA attenuated an inflammatory response triggered by ox-LDL through reducing cytokine production in RAW264.7 cells.

### Tan IIA inhibited the apoptosis of VSMCs and decreased the levels of MMP-2, MMP-9 in ApoE^–/–^ mice

**Fig. 6.**
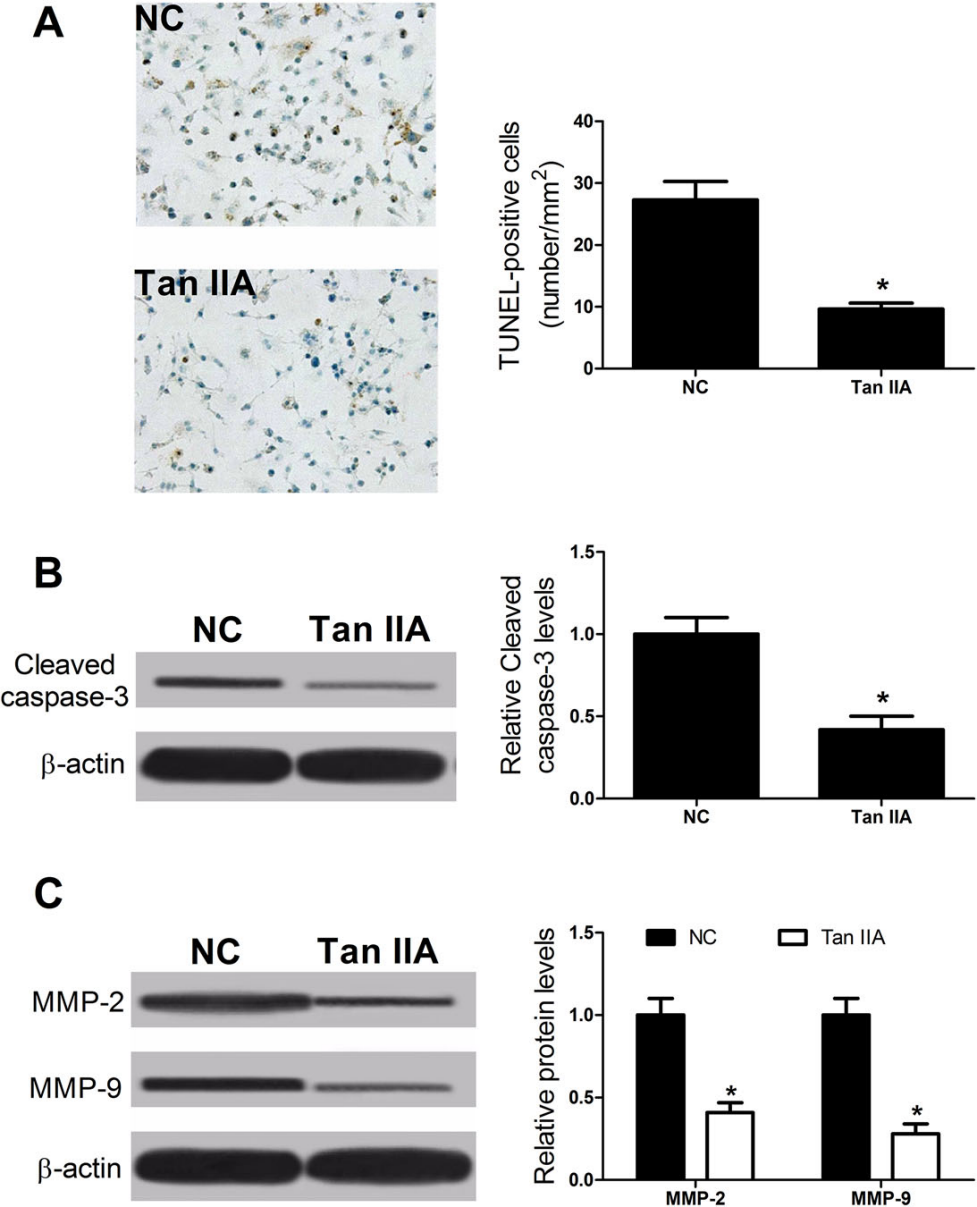
**Tan IIA reduced the apoptosis rate of VSMCs and decreased the levels of MMP-2 and MMP-9 in ApoE*^−/−^* mice.** (A) TUNEL assay showing decrease in TUNEL-positive cells in ApoE*^−/−^* mice treated with Tan IIA against to control group. (B,C) Western blots confirming the levels of cleaved caspase-3 or that the migration-related proteins MMP-2 and MMP-9 decreased when treated with Tan IIA in ApoE*^−/−^* mice, *P<0.05, error bars indicate that data were expressed as the mean±s.d. (*n*=8).

To further explore whether Tan IIA could alleviate the apoptosis of VSMCs in ApoE^–/–^ mice, VSMCs of aortic roots were estimated using TMR red. Western blot was used to detect the levels of cleaved caspase-3, MMP-2 and MMP-9. The results showed that Tan IIA could decrease the apoptosis of VSMCs in ApoE^–/–^ mice (Fig. 6A,B) and Tan IIA could also decrease the levels of MMP-2 and MMP-9 *in vivo* (Fig. 6C) in comparison with the control group.

## DISCUSSION

Illustrating the mechanisms that cause the initiation and development of atherosclerosis is vital for confirming methods to suppress its progression before it results in clinical consequences (Doran et al., 2008). Tan IIA significantly attenuated the atherosclerotic lesion in ApoE*^−/−^* mice and inhibited the formation of foam cells in atherosclerotic lesion without affecting the lipid levels of serum (Tang et al., 2011). Recently a report presented by Warnatsch et al. indicated that IL-1α, IL-1β and IL-6 were elevated in the plasma of ApoE-deficient animals after 8 weeks on high fat diet (HFD) (Warnatsch et al., 2015). In accordance with our study, Tan IIA treatment significantly reduced the atherosclerotic lesion area through inhibiting the expressions of adhesion molecules and inflammatory cytokine secretion in serum, including IL-1β, IL-6 and MCP-1, as well as TNF-α.

Many researches have indicated that lots of cell types, such as endothelial cells, lymphocytes, macrophages and smooth muscle cells (SMCs), are connected to the formation of atherosclerotic lesions (Lusis, 2000). Most atherosclerotic plaque caps consist of VSMCs, by which the stability of plaque is maintained (Clarke et al., 2006). Apoptosis of VSMCs was observed in unstable plaques (Su et al., 2015). To further confirm the protection of Tan IIA on VSMCs, its effect on of VSMCs apoptosis was investigated. Our study showed ox-LDL significantly induced apoptosis of VSMCs, while Tan IIA inhibited ox-LDL-induced apoptosis, suggesting that Tan IIA may suppress the progress of atherosclerosis in a concentration-dependent manner. Results from previous studies found that Tan IIA decreased Bax protein level in atherosclerosis plaques and moderately increased Bcl-2 protein levels, and finally induced inhibition of atherogenesis (Xu et al., 2011). Moreover, our study also indicated that Tan IIA significantly suppressed the Bax and Cleaved-caspase-3 up-regulation and reversed the Bcl-2 down-regulation in VSMCs treated with ox-LDL, which resulted in a reduced ratio of Baxl/Bcl-2, thus concluding that Tan IIA suppressed atherogenesis through regulating expression of apoptosis-related protein in VSMCs.

It is certain that cell proliferation of vascular cells is one of the mechanisms for atherosclerosis plaque growth (Andrés et al., 2012). Macrophages in the artery wall are the main cells in atherosclerosis, with the quantity and phenotype of these cells in plaques affecting disease progression (Moore et al., 2013). Our study suggested that ox-LDL induced proliferation and migration of RAW264.7 cells, while Tan IIA reversed the inductive effect of ox-LDL on RAW264.7 cell proliferation and migration. MMP-2 and MMP-9 are equal members of the MMP family. MMPs are expressed by and around forming blood vessels, and degrading extracellular matrix is a significant role of MMPs. MMP-2 and MMP-9 are also involved in local inflammatory cell infiltration in plaques and result in damage to the vessel wall and intimal defense function decline (Chen and Xu, 2014). Our data indicated that Tan IIA significantly decreased the levels of MMP-2 and MMP-9 in RAW264.7 cells pre-treated with ox-LDL.

MCP-1 and IL-6 are important cytokines in the physical and pathological processes, however IL-6 is a pleiotropic cytokine which can be both anti-inflammatory and pro-inflammatory. Moreover, pro-inflammatory cytokines TNF-α and IL-1β were also lessened by Tan IIA in RAW264.7 cells treated with LPS in a dose-dependent manner (Fan et al., 2016). In line with this, our study demonstrated that Tan IIA inhibited the ox-LDL-induced-upregulation of IL-1β, IL-6, MCP-1 as well as TNF-α in RAW264.7 cells. Thus, it is speculated that Tan IIA inhibited atherosclerosis by regulating macrophage expression of proinflammatory cytokines and chemokine in macrophages.

Our research verified that Tan IIA significantly exerted an inhibitory effect on the initiation and progression of atherosclerosis in ApoE^–/–^ mice through suppressing the expression of adhesion molecules and inflammatory cytokines secretion in serum. Tan IIA attenuates ox-LDL-induced apoptosis of VSMCs, with expression changes of Bax, Bcl-2 and cleaved caspase-3. Tan IIA suppressed the ox-LDL-induced proliferation and migration of RAW264.7 cells, accompanied by expression changes of MMP-2 and MMP-9. Additionally, our study showed that Tan IIA inhibits overexpression of TNF-a, IL-1β, IL-6, and MCP-1 in RAW264.7 cells treated with ox-LDL. However, the molecular mechanisms by which Tan IIA weakens atherosclerosis should be further investigated. In conclusion, all data suggest that Tan IIA suppresses the progression of atherosclerosis by inhibiting the apoptosis of VSMCs and the proliferation and migration of macrophages induced by ox-LDL**,** which provides new insight into the molecular mechanisms through which Tan IIA may ameliorate atherosclerosis.

## MATERIALS AND METHODS

### Preparation of ox-LDL

Native LDL was purchased from Sigma-Aldrich (Shanghai, China). For the production of ox-LDL, 200 µg/ml LDL was exposed to 20 µM CuSO_4_ in phosphate-buffered saline (PBS; Keyi, Hangzhou, China) for oxidation for 20 h at 37°C and the oxidative reactions were terminated with 40 µM butylhydroxytoluene in ethanol. Furthermore, ox-LDL was dialyzed against culture medium and sterile filtered.

### Generation of experimental atherosclerosis mice model

All animal procedures were approved by the Ethics Committee of Henan Province People's Hospital. ApoE*^−/−^* mice with a C57BL/6J background were purchased from Beijing Biocytogen (Beijing, China). All animals were kept on a regular dark/light cycle, with unrestricted access to water and standard chow (Specialty Feeds, Glen Forrest, WA, Australia) under pathogen-free conditions.

Sixteen male ApoE*^−/−^* mice at the age of 6 weeks were randomly divided into two groups of eight animals each: control group (NC) and Tan IIA (30 mg/kg) group. Mice in Tan IIA groups were gavaged with Tan IIA (30 mg/kg) suspended in 0.5% sodium carboxymethyl cellulose (CMC-Na) daily for 20 weeks, whereas mice in control groups were gavaged with 0.5% CMC-Na. After 20 weeks, the mice were killed by an i.p. injection of sodium pentobarbital (100 mg/kg; Euthatal, Sigma-Aldrich, Castle Hill, NSW, Australia).

### Cytokine detection

The levels of IL-1β, IL-6, MCP-1, and TNF-α in serum of ApoE*^–/–^* mice were determined by ELISA using the detection kit (eBioscience, San Diego, CA, USA) following manufacturer's instructions. Absorbance at 450 nm was evaluated using microplate reader. RAW264.7 macrophages were grown in 96-well plates. After treatment with ox-LDL (50 μg/ml) and Tan IIA (40 and 80 μM) for 24 h, cell-free supernatants were collected, centrifuged, and assayed for IL-1β, IL-6, MCP-1, and TNF-α by ELISA (eBioscience) according to the manufacturer's instructions.

### Tissue preparation and Oil Red O staining

After sacrifice, proximal aortae were rapidly removed from the mice, cleaned of pericardial fat under a dissecting microscope and fixed in 10% formalin. The aortas were kept at 80°C for subsequent analysis. The aortic section was immersed in 60% isopropanol (Aladdin, Shanghai, China) for 25 s and then stained with Oil Red O (Sigma-Aldrich) for 8 min. 60% isopropanol was used to remove the redundant dye for 10 s again. Sections of the aortic root were soaked in 60% isopropanol for 30 s and then stained with Oil Red O for 20 min. Washed sections were counterstained with Meyer's hematoxylin solution (Wako Pure Chemical Industries, Osaka, Japan). The entire inner surface of aortic intima and sections of aortic roots were photographed, and positive lesions were analyzed using Image J software (NIH, Bethesda, MD, USA).

### Primary aortic smooth muscle cells isolation and culture

Mouse primary VSMCs were isolated from normal aorta of male mice by combined collagenase and elastase digestion method, as previously described (Sedding et al., 2005). VSMCs were cultured in Dulbecco's modified Eagle's medium (DMEM; Invitrogen, Carlsbad, CA, USA) supplemented with 10% fetal bovine serum (FBS; GIBCO, Carlsbad, CA, USA), maintained in a 5% CO_2_ atmosphere at 37°C, and used for four to eight passages throughout this study.

### Analysis of apoptosis in VSMCs

Apoptosis was analyzed by flow cytometry assay. The cells were harvested and washed twice with cold phosphate-buffered saline (PBS). Then cells (1×10^6^) from each sample were processed with the Annexin V-FITC/PI apoptosis detection kit (BD Biosciences, San Jose, CA, USA) according to supplied protocols at indicated times. The caspase expression was assessed by flow cytometry (Becton Dickinson, Franklin Lakes, NJ, USA). In addition, TUNEL assay was used for the *in situ* cell death detection kit, TMR red, was used as described by the manufacturer (Roche Diagnostics).

### RAW264.7cell culture

The murine macrophage cell line RAW264.7 was obtained from Cell Culture Center of Chinese Academy of Medical Sciences (Beijing, China). Cells were cultured in Dulbecco's modified Eagle's medium (DMEM) supplemented with 10% HI-fetal bovine serum (FBS), 100 U/ml penicillin, and 100 μg/ml streptomycin and maintained in a 5% CO_2_ atmosphere at 37°C. The cells were treated with different concentrations of Tan IIA (at 40 and 80 μM) or ox-LDL 50 μg/ml for 24, 48 or 72 h for further experiments.

### RAW264.7 cell proliferation assay

Viable RAW264.7 cells were determined by MTT at the indicated times. Briefly, the cells were seeded at density of 2.0×10^3^ cells/well into 96-well plates (Corning Costar, Corning, NY, USA) in DMEM supplemented with 10% FBS and cultured for 24 h. Following treatment with ox-LDL (50 μg/ml) and Tan IIA (40 and 80 μM), 10 µl of 3-(4,5-dimethylthiazol-2-yl)-2,5-diphenyltetrazolium bromide (MTT; Sigma-Aldrich, USA) solution (5 mg/ml in ddH_2_O) were added to each well at indicated times (24, 48 and 72 h). The plates were incubated for a further 3∼4 h at 37°C. Intracellular formazan crystals were dissolved by the addition of 100 µl of dimethyl sulfoxide (DMSO; Sigma, St. Louis, MO, USA) to each well. Cell proliferation was determined by measuring the absorbance at 490 nm by a spectrophotometer (Multiskan MK3; Thermo Fisher Scientific, Waltham, MA, USA).

### Migration assay

The migration rate of RAW264.7 cells was determined using a Transwell^®^ chamber (Greiner; Monroe, NC, USA) with 8-µm pore filters. Tan IIA dissolved in DMEM medium containing 0.1% FBS was added in the bottom chamber. RAW264.7 cells (5×10^4^ cells/well) suspended in 100 µl of DMEM containing 0.1% BSA was added to the upper chamber. After 5 h of incubation, the lower side of the filter was washed with PBS. A cotton swab was used to remove the remaining cells on the upper side of the membrane. Cells on both side of the membrane were fixed and stained with DiffQuick staining kit (Baxter Healthcare Corp., CA, USA). Cells migrating into the lower chamber were counted in five randomly selected microscopic fields (magnification, 200×).

### Western blot

Protein samples were isolated from cultured cells using a total protein extraction kit (Kaiji Biological, Inc., Nanjing, China). The protein concentration was detected by a NanoDrop 2000 spectrophotometer (Thermo Fisher Scientific, USA). 50 µg of proteins were separated in 10% SDS-PAGE, and transferred onto polyvinylidene difluoride membrane (PVDF; Millipore, Billerica, MA, USA). Following blocking for 1 h in PBS with 0.1% Tween 20 (PBST) and 5% BSA, the membranes were incubated overnight with specified primary antibody at 4°C. PVDF membranes were washed in TBST and incubated with secondary antibody (1:5000; Santa-Cruz Biotechnology, Inc.) labeled with HRP and detected by ECL. The proteins were detected by ECL chemiluminescence detection system (Pierce, Rockford, IL, USA). The signal intensity was determined by Image J software (NIH, USA). Antibodies used in this study are Bax (1:10,000; CST Inc., Danvers, MA, USA), Bcl-2(1:5000; CST), Pro-caspase-3 (1:5000; CST), Cleaved-caspase-3 (1:5000; CST), MMP-9 (1:5000; CST), MMP-2 (1:5000; CST) and β-actin (1:2500; CST).

### Statistical analysis

Data were presented as mean±s.d. at least three experiments. The differences between different groups were analyzed using student's *t*-test or analysis of variance (ANOVA). *P*<0.05 was considered statistically significant.
