# Expression of Concern: Tanshinone IIA suppresses the progression of atherosclerosis by inhibiting the apoptosis of vascular smooth muscle cells and the proliferation and migration of macrophages induced by ox-LDL

**DOI:** 10.1242/bio.060226

**Published:** 2023-12-05

**Authors:** Baocai Wang, Zhenwei Ge, Zhaoyun Cheng, Ziniu Zhao

There are potential issues in Fig 4 in *Biol. Open* (2017) **6**, 489-495 (doi:10.1242/bio.024133).

In Fig 4B, it appears there is background duplication in the NC and 80μM Tan IIA+ox-LDL panels, and also in the ox-LDL and 40μM Tan IIA+ox-LDL panels.

We have made multiple attempts to contact all authors to request original data for the affected figures but have not received a response. Therefore, the journal is publishing this note to alert readers to our concerns.

